# Hierarchical structure in the activities of daily living and trajectories of disability prior to death in elderly Chinese individuals

**DOI:** 10.1186/s12877-021-02460-y

**Published:** 2021-10-02

**Authors:** Yaofeng Han, Jihui Xue, Wei Pei, Ya Fang

**Affiliations:** 1 State Key Laboratory of Molecular Vaccinology and Molecular Diagnostics, School of Public Health, Xiamen University, Xiang’an South Road, Xiamen, 361102 China; 2 Center for Aging and Health Research School of Public Health, Xiamen University, Xiamen, China

**Keywords:** Katz scale, Disability trajectories, Longitudinal item response theory, Older adult

## Abstract

**Background:**

The global burden of disability continues to increase. Understanding the hierarchical structure of activities of daily living (ADL) and the trajectories of disability of elderly individuals is pivotal to developing early interventions.

**Purpose:**

To determine the hierarchical structure of the ability of Chinese elderly individuals to perform ADL and further describe the trajectories of disability prior to death.

**Methods:**

Longitudinal item response theory model (LIRT) was constructed for 28,345 elderly participants in the Chinese Longitudinal Healthy Longevity Survey, in which ADL were measured using the Katz scale from 1998 to 2018, until the participants’ death. Two difficulty parameters (*κ*_−_partial and *κ*_−_total) were used in the LIRT defining the thresholds for hierarchical structure in ADL (*κ*_−_partial: no limitation to partial limitation, *κ*_−_total: partial limitation to totally limited). Disability values estimated from the LIRT were fitted to a mixed-effects model to examine the manner in which the trajectories of disability varied with different subject characteristics.

**Results:**

The findings confirmed the earliest loss in the capability to perform ADL (bathing(*κ*_-partial_ = − 1.396), toileting(*κ*_-partial_ = − 0.904)) at the level of partial limitation, with an overlap of partial and totally limited (total bathing, partial dressing, partial transferring, total dressing, partial feeding, partial continence), and finally a total loss of capability for toileting, feeding, transferring, and continence (*κ*_-total_ = 3.647). Disability trajectories varied with sex (*β* = 0.041, SE = 0.001), place of residence (*β* = 0.010, SE = 0.001), and marital status (*β* = 0.144, SE = 0.001). Females, individuals living in urban areas, and those who lived without a spouse had a poorer disability status.

**Conclusion:**

The loss in the ability to perform ADL has a hierarchical structure. Subject characteristics affect trajectories of disability in the elderly Chinese population.

**Supplementary Information:**

The online version contains supplementary material available at 10.1186/s12877-021-02460-y.

## Background

The proportion of the global population that will be older than 60 years of age will nearly double by 2050 as life expectancy increases and birth rates decline [1]. However, longer survival times do not necessarily result in extended periods of good health. With advancing age, the elderly are prone to suffer from degenerative diseases, leading to a decline in their ability to live independently [2]. Estimates predict that 55 million elderly people will be living with a disability in China by 2025 [3]. Disabilities place a heavy burden on both families and society [4]. Timely, research-based, and effective interventions that improve the utilization of care services are urgently required to mitigate the burdens caused by disability.

Scientists generally interpret disability as the loss or a limitation of a person’s ability to perform activities of daily living (ADL), an important indicator of individual health. Researchers use the Katz Index, CARS scale, and Lawton scale to assess disability [5, 6]. However, the total score of these scales may poorly discriminate individuals with a varied disability. Furthermore, a total score can also result in a floor or ceiling effect. To minimize this deficiency, the American psychometrician Lord [7] and Danish statistician Georg Rasch [8] proposed a novel test method called item response theory (IRT). In IRT, disability in the elderly can be regarded as a potential continuum, which avoids floor and ceiling effects [9]. In the different IRT models, longitudinal item response theory (LIRT) is applied to data collected over prolonged periods. The LIRT method reduces bias when estimating the trajectory of rates of decline [10]. For example, Marc et al. [11] developed a LIRT model to characterize cognition over time which was effective at capturing the multifaceted nature of cognition and its longitudinal trajectory. LIRT is also applicable in disabilities caused by progressive disease.

It has been found that ADL are hierarchical in nature [9, 12]. A number of researchers have applied LIRT and linear mixed models to capture the hierarchical structure of ADL and changes in the trajectory of disability over time by taking into account correlations across multiple measurements for each individual [9, 13]. As a general consensus, older adults tend to lose the ability to perform activities requiring strength in their lower extremities earlier than in activities requiring upper body strength [14–16]. Researchers have observed a pattern in the loss of ADL: females experience an initial loss in their ability to walk independently outside the home, followed by the inability to independently groom, bathe, dress, toilet, and feed themselves. Males experience a similar pattern, except that the inability to dress occurs second [17]. In addition, trajectories of disability change dynamically over time. Although controversial, a number of studies report that change in disability is sex-specific and influenced by level of education.

The present study aimed to use LIRT and a mixed-effects model to study the hierarchical structure of ability to perform ADL and the trajectories of disability of individuals over 60 years old in China. The outcomes will constitute a reference for the development of effective interventions for elderly people living with disability.

## Method

### Study population

The Chinese Longitudinal Healthy Longevity Survey (CLHLS) began in 1998 with data collection continuing in 2000, 2002, 2005, 2008–2009, 2011–2012, 2014, 2017–2018, representing eight waves in total. Investigations were conducted randomly in approximately half of the cities/counties in 23 of 31 Chinese provinces, the total population of which represented approximately 85% of the total population of China. Due to the mixed longitudinal design of CLHLS, only one-third of subjects from each wave had been studied in the previous wave, with new recruits representing the remaining participants. From 1998 to 2014, 71,130 participants were interviewed, including 16,582 centenarians, 23,207 nonagenarians, 20,135 individuals aged 65–79, and 11,206 adults aged 35–64. A total of 12,411 new subjects were included in the cohort in 2018. A more detailed analysis has been made of these [18]. The project has collected the data of individual demographic characteristics, lifestyle, physical and mental health, and survival status [19]. The present research protocol was approved by the Duke University Institutional Review Board (Pro00062871) and Peking University Biomedical Ethics Committee (IRB00001052–13074). All methods were conducted in accordance with relevant guidelines and regulations, and all participants or their legal representatives gave written informed consent.

**Fig. 1:**
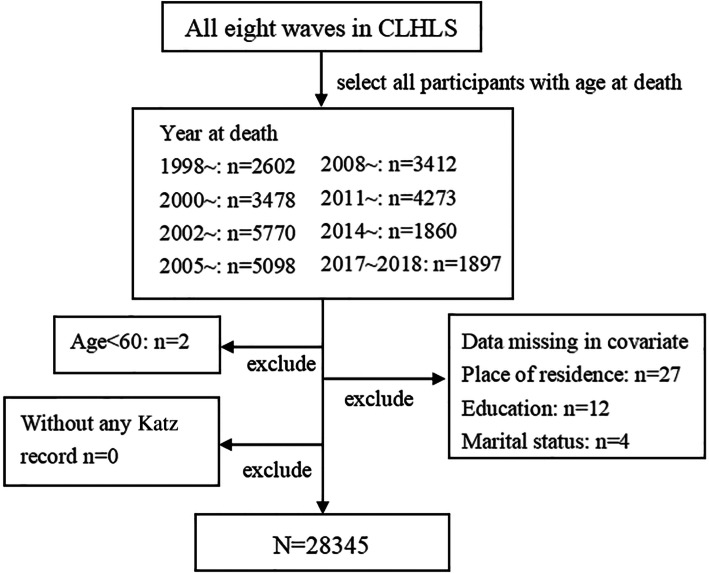
Data processing process

To fully reflect the natural developmental processes of disability status prior to death, a dynamic model was constructed that spanned all eight waves in which participants older than 60 years of age were selected, with the exact age at death, including those who had at least one Katz measurement available for inclusion in the analysis. We excluded all individuals with incomplete data describing subject characteristics. The data processing process was depicted in Fig. 1.

### Disability status assessment

The present study used the Katz scale to assess the ability of individuals to perform activities of daily living, which the Katz scale evaluates, including bathing, dressing, transferring, feeding, toileting, and continence, using a three-point scale (0: no limitation, 1: partial limitation, 2: totally limited) for task performance. The Chinese version, which has been extensively tested in pilot interviews, has yielded reliable and valid responses [20]. Participants refusing or declining to answer a question were handled as missing cases.

### Model construction for the hierarchical structure of ADL

In light of the ordered categorical responses of the Katz scale, a graded response model (GRM), proposed by Samejima [21] was selected as a generic IRT building block to relate each test item with disability status. In the GRM model for hierarchical reactions, every item had three parameters: (I) Two difficulty parameters (*κ*_-partial_ and *κ*_-total_) defining the thresholds for change (from no limitation to partial limitation and from partial limitation to totally limited, corresponding to *κ*_*-*partial_ and *κ*_*-*total_, respectively). For example, bathing_-partial_ indicated the threshold from no limitation to partial limitation for bathing. In the disability severity continuum, items with a small *κ* represent activities that elderly individuals are more likely to lose the ability to perform. In the present study, difficulty parameters reveal the hierarchical structure of the ADL. (II) An item with a higher discrimination parameter (*α*) is one for which individuals could more closely be positioned at the correct disability level.

To achieve the dual goals of identification of difficulty and discrimination parameters of the Katz scale and assessment of the decline in the ability to perform ADL, we chose an LIRT model for data analysis [11]. Each individual (*s*) response score of a specific item (*i*) at a given time (*t*) was recorded as *Y*_*s*, *t*, *i*_, while the corresponding disability status of an individual was *θ*_*s,t*_ (every six items corresponding to the same *θ*, with larger *θ* values indicating a more serious disability status). In the present study, the response category of every item was *K* = 3, with a cumulative probability expressed as *P*_*s*, *t*, *i*, *K*_ = 1.

$$ {P}_{s,t,i,k}=P\left({Y}_{s,t,i}\le k\mid {\theta}_{s,t}\right)-P\left({Y}_{s,t,i}\le k-1\mid {\theta}_{s,t}\right) $$

$$ Logit\left({p}_{s,t,i,k}\right)=\log \left(\frac{p_{s,t,i,k}}{1-{p}_{s,t,i,k}}\right)={K}_{i,k}-\alpha i{\theta}_{s,t}k=1,2,3 $$

where *K*_*i*, *k*_ represented the difficulty parameter of item *i*, and *α*_*i*_ represented the discrimination parameter of item *i*. *k* corresponded to the selected category of the individual. The longitudinal aspect of the model was captured by setting *θ*_*s*, *t*_ = *γ*_0, *s*_ + *γ*_1, *s*_ × *t*. *γ*_0, *s*_ and *γ*_1, *s*_ represented the intercept and slope of disability status. The mean value of *γ*_1, *s*_ was described by *μ*, representing the mean decline of ADL over time. *γ*_1, *s*_ was modeled as ${\gamma}_{1,s}={\gamma}_{1,s}^{\prime }+{Z}_s\beta$. *Z*_*s*_ represented each characteristic of a subject, including sex, age, years of education, place of residence, and marital status, *β* referred to the regression coefficients of *Z*_*s*_, while ${\gamma}_{1,s}^{\prime }$ was the intercept of *γ*_1, *s*_. Age and years of education were zero-centered and standardized for model fitting.

The model was cast within a Bayesian framework and then implemented using a Markov Chain Monte Carlo (MCMC) method. The posterior 95% credible interval for each parameter was displayed with posterior credible intervals (Q2.5, Q97.5). A weak information prior distribution was adopted in all parameters except *γ*_0, *s*_ [22], which used an independent *N* (0,1) prior distribution. The slope *γ*_1, *s*_ selected a hierarchical prior distribution.

### Trajectories of disability

To determine trajectories of disability, the time between interview and death was used as the independent variable (*t*) and the disability level (*θ*) of the elderly individual, obtained using the aforementioned LIRT, represented the dependent variable (scale to [− 3,3]). A mixed-effects model [23] was used to analyze changes in function over time. The model is shown below:

$$ {\theta}_{s,t}={\eta}_{1s,}+{\eta}_{2s}{t}_s+{e}_{s,t}\bullet {e}_{s,t}\sim N\left(0,{\sigma}^2\right) $$

$$ {\displaystyle \begin{array}{c}{\eta}_{1s={\beta}_1+{b}_{1s}}\\ {}{\eta}_{2s={\beta}_2+{b}_{1s}}\end{array}}\left({b}_{1s},{b}_{2s}\right)\sim MVN\left(0,\varSigma \right) $$

where *θ*_*s*, *t*_ was the elderly individual’s potential disability at time *t*, *η*_1*s*_ was the intercept when *t* was equal to 0, while *η*_2*s*_ reflected changes in *θ*_*s*, *t*_ over time. The parameter vector (*b*_1*s*_, *b*_2*s*_) was multivariate normally distributed with mean of zero and a 2 × 2 variance-covariance matrix *Σ*. Using this process, *t* was considered level one, *s* as level two, and all subject-specific covariates (sex, place of residence, marital status, sex*place of residence, age, years of education, sex*marital status) were included in the model as adjustment factors.

The LIRT methodology was performed using OpenBUGS software while calculations for the mixed-effects model were conducted using MLwiN software. Pre and post-data processing were completed in the R_3.5.3_ environment. OpenBUGS software used thin = 1 and 1000 iterations after a 4000 burn-in.

## Results

### Subject characteristics

The exact age at death was recorded for 28,390 individuals for which there was at least one Katz record. After excluding 45 individuals with incomplete covariate data (a missing ratio of 0.15%), 28,345 participants were included in the study. The median elapsed time between the initial and final visit was 3 years (range: 0–19 years). Females accounted for approximately 60% of the study population (Table 1). Mean age was 91.3 years, ranging from 60 years to 122 years. Regarding years of education, 70.7% of participants had never been to school, and more than 90.6% had a low level of education - less than 5 years. The majority of participants (63.1%) lived in rural areas, the remaining 36.9% residing in urban areas. Approximately 80% of the participants did not live with a spouse.

**Table 1:** Baseline characteristics of the study population

	No.	%	Mean (SD)
Gender	11,280		
Male		39.8%	
Female	17,065	60.2%	
Place of residence			
Urban	10,448	36.9%	
Rural	17,897	63.1%	
Current marital status			
With spouse	5444	19.2%	
Without spouse	22,901	80.8%	
Age			
Mean (SD)			91.3(9.6)
Years of education			
Mean (SD)			1.4(2.9)

### Discrimination parameters

Table 2 displays the posterior distributions of the discrimination parameters. The posterior means of ADL items were ranked from toileting, transferring, dressing, feeding, bathing, and continence (range: 1.125 to 4.124).

**Table 2:** Posterior means and 95% credible intervals of the discrimination parameters for items in Katz scale

	Mean	SE	Q2.5	Q97.5
Bathing	1.291	0.003	1.261	1.317
Dressing	2.877	0.007	2.810	2.917
Toileting	4.124	0.015	3.984	4.286
Transferring	3.682	0.007	3.565	3.783
Continence	1.125	0.003	1.077	1.168
Feeding	2.137	0.004	2.071	2.197

### Difficulty parameters

The hierarchical structure of declining ability to perform ADL, provided by the estimated difficulty parameters, is presented in Table 3. Firstly, with a range between − 1.396 and 3.647, we confirmed that loss in the ability to perform ADL began with bathing_-partial_ and ended with continence_-total_. In particular, bathing_-partial_ (*κ*_-partial_: − 1.396 (standard error (SE), 0.003)) and toileting_-partial_ (*κ*_-partial_: -0.904 (SE, 0.006)) were observed in succession. The last item for which the elderly required partial assistance was continence. Bathing was the first task that was restricted at the level of totally limited (*κ*_-total_: -0.374 (SE, 0.003)). Finally, at the end of the structure, feeding (*κ*_-total_: 3.440 (SE, 0.004)), transferring (*κ*_-total_: 3.454 (SE, 0.008)), and continence (*κ*_-total_: 3.647 (SE, 0.013)) were totally limited.

**Table 3:** Posterior means and 95% credible intervals of the difficulty parameters (*κ*) for items in Katz scale

	Mean	SE	Q2.5	Q97.5
Bathing_-partial_	−1.396	0.003	−1.433	−1.364
Toileting_-partial_	−0.904	0.006	−0.953	− 0.843
Bathing_-total_	−0.374	0.003	−0.406	− 0.366
Dressing_-partial_	−0.277	0.004	−0.315	− 0.238
Transferring_-partial_	−0.053	0.004	−0.082	− 0.017
Dressing_-total_	0.482	0.004	0.441	0.520
Feeding_-partial_	0.917	0.005	0.884	0.962
Continence_-partial_	1.371	0.003	1.324	1.409
Toileting_-total_	3.348	0.009	3.238	3.478
Feeding_-total_	3.440	0.004	3.351	3.521
Transferring_-total_	3.454	0.008	3.325	3.562
Continence_-total_	3.647	0.013	3.535	3.739

### Effect of subject-specific characteristics on the rate of decline

Table 4 displays the regression coefficients of the subject characteristics for individual slopes *γ*_1, *s*_. Sex, years of education, place of residence, marital status, and age displayed significant associations with slope values. The value can only be interpreted in relative terms (not the risk of disability), such that males, older age, fewer years of education, urban residence, and living without a spouse was associated with a faster decline. The posterior mean of the slope parameter *μ* was 0.857 (2.5 and 97.5% quantiles [0.824, 0.891], SE = 0.003), indicating that individual disability increased by 0.857 units per year.

**Table 4:** Regression coefficients for the individual slopes *γ*_1, *s*_ in LIRT

	Mean	SE	Q2.5	Q97.5
Male/Female	0.102	0.001	0.089	0.117
Baseline Age	0.889	0.000	0.883	0.894
Urban/Rural	−0.591	0.003	−0.614	− 0.566
Years of Education	−0.730	0.002	−0.743	− 0.718
Marital Status	0.056	0.003	0.021	0.078

Note: The binary covariates were coded as sex [0: male, 1: female], place of residence [0: urban, 1: rural], marital status [0: with a spouse, 1: without a spouse]. Years of education and baseline age were zero-centered and standardized for model fitting

### Trajectories of disability in the mixed-effects model

**Fig. 2:**
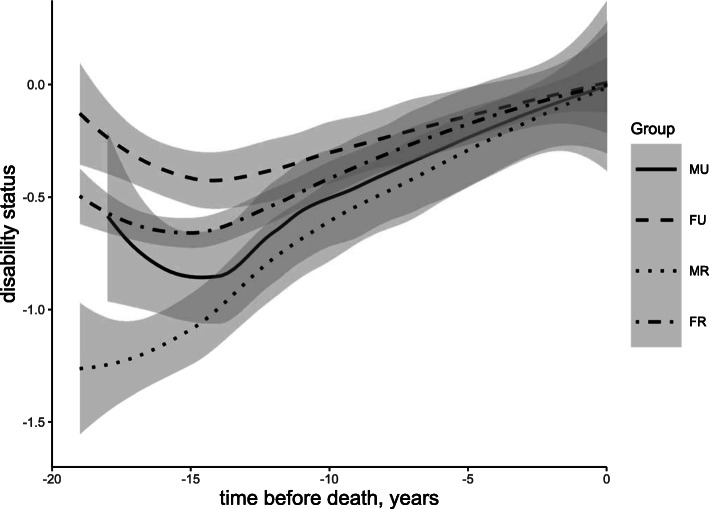
Nineteen-year mean trajectories of disability preceding death grouped by gender*place of residence. Note: MU means males living in urban, FU means females living in urban, MR means males living in rural, FR means females living in rural. Adjusted for age, marital status and years of education

**Fig. 3:**
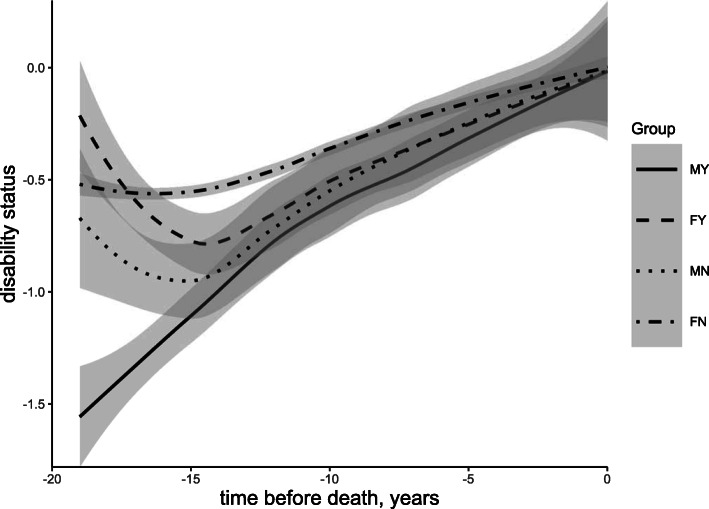
Nineteen-year mean trajectories of disability preceding death grouped by gender*marital status. Note: MY means males living with a spouse, FY means females living with a spouse, MN means males living without spouse, FN means females living without spouse. Adjusted for age, place of residence and years of education

Trajectories of disability varied with sex (*β* = 0.041, SE = 0.001), place of residence (*β* = 0.010, SE = 0.001), and marital status (*β* = 0.144, SE = 0.001). Females, individuals residing in urban areas, and those living without a spouse had a poorer disability status. Further cross-group analysis in Fig. 2 indicates that, compared with males living in rural areas (mean = − 0.219, SE = 0.001), the disability status of males living in urban areas (mean = − 0.163, SE = 0.001), females living in rural areas (mean = − 0.124, SE = 0.001), and females living in urban areas (mean = − 0.085, SE = 0.001) declined progressively. As displayed in Fig. 3, compared with males living with a spouse (mean = − 0.265, SE = 0.001), the disability status of males living without (mean = − 0.249, SE = 0.002), females living with a spouse (mean = − 0.164, SE = 0.001), and females living without a spouse (mean = − 0.099, SE = 0.001) became sequentially worse. Detailed disability status and cross-group characteristics are displayed in Supplementary Table S1. After a brief fluctuation in the 15–19 years prior to death, the function displays a deterioration from the 15th year.

## Discussion

We studied the natural history of disability over the last 19 years of life in the CLHLS cohort. We provided a synthetic hierarchy of the Katz scale’s rating of ADL performance and described the trajectories of disability preceding death. As is well known, greater severity of disability in the elderly results in additional burden on caregivers [24]. Therefore, preventing or slowing the occurrence of disability in the elderly should be a key national focus. Unfortunately, few international studies have examined the natural history of disability in the elderly. Governments routinely advocate preventive strategies for health problems, and as such, geriatric medicine must evolve to intervene at an earlier stage of the disability process to be more effective. To date, a number of research studies have demonstrated a positive effect of primary prevention on dependence morbidity [25–27]. For the elderly, disability is a primary dimension of health and function, and it acts as an indicator or guideline for developing health policies for this age group [28]. The present study provides foundational evidence upon which to formulate early intervention policies for preventing disability in the elderly.

### Item discrimination

According to the guidelines of Baker [29] for a normal ogive model, the discrimination value for continence was moderate, with all other items very high. Poor discrimination refers to a task or activity (i.e., scale items) that proves unresponsive to changes in a particular person’s level of disability. In the present research, the discrimination values of all items were greater than 0.3 [30], demonstrating that the Katz scale is applicable to the Chinese elderly population as a whole. Similarly, other researchers have found the same ranking of parameters (toileting > transferring > dressing) in a two-parameter IRT model [31, 32]. In previous calculations of parameters between ADL items, it was generally believed that discrimination for toileting, dressing, and bathing was greater [33, 34]. However, in the present study, there was a moderate discriminatory effect for bathing. Saliba et al. [34] divided elderly people aged over 65 years into two groups (65–84 years and ≥ 85 years) and found that bathing displayed a smaller degree of discrimination in the older age group when model fitting. The very high age range in this study may be the reason that bathing could not more effectively distinguish different levels of disability.

### Hierarchical structure of ADL

The continuum of declining ability to perform ADL began with bathing_-partial_ and ended with continence_-total_. As noted in previous publications [17, 35–37], bathing was the first ADL to deteriorate, defined by Katz et al. as the threshold of disability. In the present study, the difficulty parameter between bathing_-partial_ and bathing_-total_ was only 1.022, confirming that bathing was the first ADL to be lost among the elderly. Researchers have reported that bathing is the first ADL that both elderly Americans and Chinese have difficulty in performing [14]. However, the bathing task was informative only in the range of low capability, so partial and total limitation in bathing occurred when the elderly were only slightly disabled.

The second ADL to be lost was toileting_-partial_. Of the six items explored here, the behavior of the toileting item was peculiar. The distance on the continuum between its partial and total limitation thresholds was considerably higher (4.252) than the other activities (ranging from 0.759 (dressing) to 3.507 (transferring)). Furthermore, its discriminative capability was very high, indicating that the item correctly discriminates between individuals at the two levels of disability.

### Factors affecting deteriorating ability status

The Bayesian methodology allowed longitudinal slope estimates to remain vague for subjects with little (or no) follow-up time. We fitted our model on all participants and concluded that males, greater age, fewer years of education, living in rural areas or without a spouse were associated with a faster deterioration in the ability to perform ADL. We discovered that older females had a higher level of disability, but their functional capabilities deteriorated at a slower rate. As a result, there are a larger proportion of dependent women within the elderly population in China. Education has always been considered a factor that slows aging and decline in ability [38–40]. The probable reason is that people with lower levels of education pay less attention to their physical health, not to mention the prevention of chronic disease. In addition, this phenomenon is more common in rural areas. It has long been argued that marital status, as a defining feature of the social environment, affects an individual’s risk of disability [41]. Marital status is significantly associated with physical disability [42, 43], and marital closeness moderates the negative psychological effects of high levels of disability on depression, anxiety, and self-esteem [44].

### Trajectories of disability

The estimated trajectories of disability highlighted that the extent of disability became progressively serious as death approached. Trajectories of disability at the end of life are quite variable [45, 46]. In the mixed-effects model, females, living in urban areas, and living without a spouse had worse ADL status. Interestingly, disability status was better in rural areas. It may be that rural medical services are relatively inaccessible, and older adults become frailer at a younger age dying earlier, so the remaining elderly are in better condition [47]. Overall, differentiating the expected trajectories and related needs would help tailor strategies and programs to improve elderly care prior to death.

### Strengths and weaknesses

The innovative feature of the present study was that it relied on a longitudinal analysis of long-term follow-up data from a substantial sample of the general elderly population. Moreover, the LIRT and mixed-effects models allowed a capture of the multifaceted nature of disability. The LIRT also allowed us to estimate item and disability distribution parameters, as the data were from a longitudinal cohort study. In addition, the correlation between the subject-specific characteristics and the slope of deterioration was embedded in the same model.

Three limitations of this study should be noted. It has been suggested that instrumental ADL (IADL) and ADL have a hierarchical relationship, with older adults first declining in IADL functionality [48, 49]. However, there were no IADL items investigated in this survey, preventing exploration of the hierarchy of ADL and IADL disability. We found that OpenBUGS is a time-consuming statistical software package when adding too many covariates in the process of implementing MCMC. Comorbidities may also affect disability of the elderly. As an exploratory analysis, we have not considered chronic diseases for the time being, but we will look for more efficient software to research the influence of chronic diseases and other influencing factors in subsequent studies. Unavoidably, there may also have been investigation bias and survivor bias during the follow-up period.

## Conclusion

In conclusion, toileting and bathing are promising domains for detecting early signs of disability in the elderly Chinese population. At the same time, toileting and transferring are more discriminative than other ADL items. LIRT methodology and the mixed-effects model, as applied here in an elderly population, are suitable methods to jointly capture the multifaceted nature of disability and its rate of change. As a result, we found that males, those of older age, fewer years of education, living in rural areas, and living without a spouse often decline faster in their ability to perform ADL. However, females, individuals who live in urban areas and those without a spouse had a lower ability to perform ADL. Therefore, we recommend a reasonable allocation of health resources toward mitigating declining ability and encourage the widowed elderly to engage in more social activities. Furthermore, health interventions are required to address deficits in the home bathing environment, especially in developing countries such as China.

## Supplementary Information

**Additional file 1: Table S1.** Disability status of the elderly with cross-group characteristics.

## Data Availability

The raw data is available online (https://opendata.pku.edu.cn). Researchers can obtain these data after submitting a data use agreement to the CLHLS team.

## Abbreviations

ADL: Activities of daily living
IADL: Instrumental activities of daily life
IRT: Item response theory
LIRT: Longitudinal item response theory
CLHLS: Chinese longitudinal healthy longevity survey
GEM: Graded response model
