# Correction to: Hierarchical structure in the activities of daily living and trajectories of disability prior to death in elderly Chinese individuals

**DOI:** 10.1186/s12877-022-02856-4

**Published:** 2022-03-22

**Authors:** Yaofeng Han, Jihui Xue, Wei Pei, Ya Fang

**Affiliations:** 1 State Key Laboratory of Molecular Vaccinology and Molecular Diagnostics, School of Public Health, Xiamen University, Xiang’an South Road, Xiamen, 361102 China; 2 Center for Aging and Health Research School of Public Health, Xiamen University, Xiamen, China

**Correction to: BMC Geriatr 21,522 (2021)**

**https://doi.org/10.1186/s12877-021-02460-y**

After publication of this article [1], the authors reported that the term 'MMSE' should have read 'Katz', in all 3 occurrences.

The original article [1] has been updated.
