# Supplementary material for: Hierarchical structure in the activities of daily living and trajectories of disability prior to death in elderly Chinese individuals
Source: BMC Geriatr. 2021 Oct 2;21:522. doi: 10.1186/s12877-021-02460-y (PMC8487510; doi:10.1186/s12877-021-02460-y)
Supplement: Supplementary file 1 — Additional file 1: Table S1. Disability status of the elderly with cross-group characteristics. [file 12877_2021_2460_MOESM1_ESM.doc]

**Table S1** Disability status of the elderly with cross-group characteristics

|  | **marginal Mean** | **SE** | ***F*** | ***P*** |
| --- | --- | --- | --- | --- |
| **Gender & place of residence** |  |  |  |  |
| Male lived in Urban | -0.139 | 0.001 | 574.150 | <0.0001 |
| Female lived in Urban | -0.111 | 0.002 |
| Male lived in Rural | -0.184 | 0.001 |  |  |
| Female lived in Rural | -0.150 | 0.001 |  |  |
| **Gender & marital status** |  |  |
| Male lived with spouse | -0.171 | 0.001 |  |  |
| Female lived with spouse | -0.132 | 0.002 | 63.374 | <0.0001 |
| Male lived without spouse | -0.153 | 0.001 |
| Female lived without spouse | -0.129 | 0.001 |  |  |

Note：Adjust for age and education levels.
